# Sexual behaviors among methadone maintenance patients in a mountainous area in northern Vietnam

**DOI:** 10.1186/s13011-017-0123-4

**Published:** 2017-08-25

**Authors:** Victoria L. Boggiano, Huong Lan Thi Nguyen, Long Hoang Nguyen, Tho Dinh Tran, Hung Van Nguyen, Huong Thi Le, Hai Quan Le, Canh Dinh Hoang, Cuong Tat Nguyen, Bach Xuan Tran, Carl A. Latkin, Nabil Zary, Thuc Minh Thi Vu

**Affiliations:** 1 Berkeley School of Public Health, University of California, Berkeley, California USA; 2 Institute for Global Health Innovations, Duy Tan University, Da Nang, 550000 Vietnam; 3 School of Medicine and Pharmacy, Vietnam National University, Hanoi, Vietnam; 4 Authority of HIV/AIDS Control, Ministry of Health, Hanoi, Vietnam; 5 Institute for Preventive Medicine and Public Health, Hanoi Medical University, Hanoi, Vietnam; 6 Tuyen Quang Provincial AIDS Center, Tuyen Quang, Vietnam; 7 Department of Hepatobiliary Surgery, Vietnam-Germany Hospital, Hanoi, Vietnam; 8 Johns Hopkins Bloomberg School of Public Health, Baltimore, MD USA; 9 Lee Kong Chian School of Medicine, Nanyang Technological University, Singapore, Singapore; 10 Department of Immunology and Allergy, National Otolaryngology Hospital, Hanoi, Vietnam

**Keywords:** Methadone, MMT, Sexual behaviors, Vietnam

## Abstract

**Background:**

Methadone maintenance treatment (MMT) improves patients’ ability to access HIV-related services and reduces needle sharing and other risky HIV-related behaviors. However, patients may continue to engage in risky sexual practices. In this study, we evaluate sexual behaviors of MMT patients in a mountainous province in Northern Vietnam.

**Methods:**

We explored the health status, MMT and substance use history, and sexual practices of 241 male MMT patients in Tuyen Quang province. Health status was investigated using the EuroQOL-5 Dimensions-5 Levels (EQ-5D-5 L). Multivariate logistic regression was employed to assess associated factors.

**Results:**

Most patients (66.4%) reported having at least one sexual partner within the previous twelve months. Most of these partners were spouses or primary partners (72.6%). About 8.3% of patients had casual partners, and 5.8% had visited sex workers; of those who engaged in casual sexual relationships, 90.9% reported using condoms. Current drug use and living in a remote area were associated with an increased odd of having two or more sexual partners, while anxiety or depression was associated with lower odds.

**Conclusion:**

This study highlights a low proportion of having sexual risk behaviors among MMT patients in Vietnamese mountainous settings. Integrating education about safe sexual practices into MMT services, along with providing medical care and ensuring methadone treatment adherence, is an important component in HIV risk reduction for these patients who were at risk of unsafe sexual practices.

## Background

Methadone is widely used for opioid addiction treatment, due to its high effectiveness on reducing the frequency of illicit drug use and injection [1]. However, the impact of methadone maintenance treatment (MMT) on risky sexual behaviors has been debated. Empirical evidence about the sexual behaviors of MMT patients have appeared in a variety of contexts with different populations [1], including former MMT patients [2], cocaine users [3, 4], HIV positive patients [5] and female sex partners [6]. While several studies indicate the positive effects of MMT on increasing the protective sexual behaviors among the patients [1, 7], others reported no impact on condom use or a lower level of condom use among patients on MMT compared to their non-MMT counterparts [8]. Furthermore, in some settings, patients on MMT appear to have poor understanding of safe sex practices and HIV prevention methods [5]. Therefore, more data about sexual behaviors among MMT patients is needed to develop interventions in this population.

Vietnam has a concentrated HIV/AIDS epidemic [9] with more than 250,000 individuals living with HIV/AIDS [10]. Vietnam has seen a rise in injection drug use over the past decade [11] and the leading cause of new HIV infections is via needle sharing [10]. However, currently, there is an increasing trend nationwide of HIV being spread by risky sexual behavior in most-at-risk populations such as sex workers, men who have sex with men, and injection drug users [12].

The sexual behaviors among people who inject drugs (PWID) in Vietnam has been extensively studied [8, 13]. Authors have found that injection drug users are likely to engage in high risk sexual practices such as having sex with sex workers [8, 13] and infrequent condom use [13]. PWID also put their female partners at risk of HIV and other STDs [9, 14].

MMT initiatives have begun to take root across Vietnam in an effort to reduce injection drug use and high-risk behaviors namely sexual risk behaviors [15]. The risk taking behaviors of patients on MMT in various rural and urban settings in parts of Vietnam have been studied in the past [16]. In addition, individuals living in mountainous parts of Vietnam, which are hard-to-reach areas, have been found to have a higher prevalence of a range of health problems [17]. To date, however, little is known about the sexual behaviors of patients after receiving MMT in mountainous regions of Vietnam. In this study, we aimed to profile sexual behaviors of MMT patients in Tuyen Quang province, a mountainous region in northern Vietnam.

## Methods

### Study setting and sampling method

A cross-sectional study was conducted from May to August 2016 in Tuyen Quang, a mountainous province in Vietnam with a population of 760,289 people spreading across 5867.9 km^2^ [18]. There were two MMT clinics involved in the study: Tuyen Quang City and Son Duong clinics. The former represents an urban area in Tuyen Quang province, while the latter reflects a more remote and rural area of the region. There were 274 patients currently managed in these two clinics.

Our target population was drug users who currently received oral methadone treatment. The study eligibility criteria included: 1) presenting at clinics during study periods; 2) being 18 years old or above; 3) having the capacity to answer a questionnaire; and 4) agreeing to participate. Patients were excluded if they: 1) had cognitive impairment or were unable to answer questionnaire and 2) refused to enroll into the study. When first meeting the patients, we invited all eligible people to enroll in this study and give written informed consent if they agreed to participate. We invited them to a private room of the clinic to ensure the confidentiality and a pleasant atmosphere for the interview. Because we only interviewed patients with a questionnaire, the respondents did not suffer any adverse effects.

Due to a small number of patients in two clinics, we intended to recruit all of patients. However, some patients did not accept to participate because of several reasons such as 1) being uncomfortable; 2) feeling not private and 3) having busy work. A convenience sample of 241 patients was enrolled in the study. The response rate was 80–90% across sites. The duration of MMT among patients ranged from 1 to 38 months.

### Measurements and instruments

Face-to-face interviews were conducted by well-trained interviewers including health staff working at the Tuyen Quang Center for Preventive Medicine and master students of public health at the Hanoi Medical University.

### Socioeconomic characteristics

Socioeconomic characteristics included age, gender, education level, marital status, employment status, ethnics, religions and household monthly income.

### Sexual behaviors

To assess sexual activities, patients were asked about how often they had sexual intercourse, the number of sexual partners they had in the last 12 months, the types of sexual partner(s) they had (primary, casual, commercial) in the last 12 months, and condom use during the last sexual encounter with each type of partner. These measures have been successfully applied in the previous studies in different populations namely MMT patients and clients in HIV testing and counseling services (HTC) [16, 19, 20]. Patients had sexual risk behaviors when they had two or more sexual partners and did not use condom with their casual partners/ sex workers.

### Health status

Health-related quality of life (HRQOL) was measured by using the EuroQol - five dimensions - five levels (EQ-5D-5 L) instrument. Five domains are included in the descriptive system: Mobility, Self-care, Usual activities, Pain/Discomfort and Anxiety/Depression. Within each domain, five responses were available: no problems, slight problems, moderate problems, severe problems, and extreme problems. This gave a matrix of 3125 health states with respective single indexes. To compute those indexes, the interim scoring for EQ-5D-5 L from cross-walk value set of Thailand was utilized due to the unavailability of Vietnamese population’s preference [15, 21]. The Vietnamese version of EQ-5D-5 L was translated and adopted as well as validated elsewhere [15]. Furthermore, we also asked patients to report their sero-HIV status and whether they currently received antiretroviral treatment or not.

### Substance use

Patients were asked to report their frequency of using alcohol (never/ monthly/ weekly/ 2–3 times per week/ 4 times per week or more). Participants that had smoked at least 100 cigarettes in their entire lives and had smoked within the last 30 days were classified as current cigarette smokers. We also collected information on participants’ history of intravenous drug abuse (IVDA) and current illicit drug use via self-reported items. We also investigated the duration of MMT among patients.

### Statistical analysis

Data was analyzed by using STATA software version 12.0 (Stata Corp. LP, College Station, United States of America). Mann-Whitney test was used to test the difference of the percentage of using condom in the last 12 months between two sides (with non-normal distribution). Chi-squared test was applied to measure the differences of other categorical variables between urban and remote areas. A *p*-value <0.05 was considered statistically significance.

Multivariate logistic regression was used to examine the associated factors with variables “having two sex partners or more”. Due to the small number of participants having sexual risk behaviors without condom, we decided to not use the variable “not using condom in the last sexual intercourse with casual partners/ sex workers” as an outcome in the regression model. The potential related factors included an “a priori” defined set of variables such as socio-demographics (age, education, marital status, employment, income quintiles); health-related quality of life and HIV status; substance abuse (current smoker, current drug use, ever drug injection, using alcohol). A stepwise backward selection strategy was applied along with multivariate logistic regressions to have reduced models. This strategy used threshold with the log-likelihood ratio test to have predictors with *p*-values of <0.2 included.

## Results

A total of 241 participants, all males, completed the survey. The mean age of participants was 40.4 (SD = 7.5) years. About 8.1% completed high school. Most (77.6%) were employed in work other than farming. More than half lived with their spouse/partner (62.3%) and an additional 22.9% were single. (Table 1).

**Table 1:** Characteristics of respondents (*n* = 241)

Characteristics	Urban area		Remote area		Total	
	n	%	n	%	n	%
Age group						
< 30	8	4.8	5	6.8	13	5.4
30–40	72	43.1	31	41.9	103	42.7
40–50	65	38.9	25	33.8	90	37.3
> =50	22	13.2	13	17.6	35	14.5
Gender						
Male	167	100.0	74	100.0	241	100.0
Education Attainment						
< High school	71	42.8	40	57.1	111	47.0
High school	78	47.0	28	40.0	106	44.9
> High school	17	10.2	2	2.9	19	8.1
Marital status						
Single	45	27.1	9	12.9	54	22.9
Live with spouse/partner	97	58.4	50	71.4	147	62.3
Divorced/widow	24	14.5	11	15.7	35	14.8
Job						
Unemployed	9	5.4	6	8.6	15	6.4
Worker/Famer	9	5.4	29	41.4	38	16.1
Self-employed	87	52.4	25	35.7	112	47.5
Others	61	36.8	10	14.3	71	30.1

Self-reported health status of participants is presented in Table 2. Few participants (10–20%) reported having problems with mobility, self-care, usual activities, or pain/ discomfort. A slightly higher proportion (25.9%) of patients reported anxiety/depression. The majority (97.4%) reported ever injecting drug use, but a small minority reported current drug use (13.4%) or using alcohol (33.1%). The overall EQ-5D score of participants was 0.90 (SD = 0.20). The mean duration of MMT was 15.3 (SD = 8.7) months. Approximately one-fourth (25.5%) of patients had HIV positive.

**Table 2:** Health related quality of life and risk behavior among respondents

Characteristics	Urban area		Remote area		Total	
	n	%	n	%	n	%
EQ5D5L						
Having problem in mobility	25	15.1	8	11.4	33	14.0
Having problem in self-care	21	12.7	4	5.7	25	10.6
Having problem in usual activities	29	17.5	5	7.1	34	14.4
Pain/Discomfort	39	23.5	8	11.4	47	19.9
Anxiety/Depression	52	31.3	9	12.9	61	25.9
Having HIV positive	32	19.9	27	38.6	59	25.5
Substance use						
Ever injecting drug	162	98.2	66	95.7	228	97.4
Current illicit drug use	21	13.0	10	14.3	31	13.4
Current cigarette smokers	129	79.6	45	66.2	174	75.7
Using alcohol	53	31.9	24	35.8	77	33.1
Receiving antiretroviral treatment	28	17.1	25	36.2	53	22.8
	X	SD	X	SD	X	SD
EQ-5D index	0.9	0.2	0.9	0.2	0.9	0.2
MMT duration	16.4	9.2	12.7	6.7	15.3	8.7

Table 3 describes the sexual behavior of the respondents. Among 223 respondents, the majority of respondents (66.4%) reported having one sexual partner in the last twelve months. A small minority reported having two partners (9.9%) or more than two partners (4.5%) in the last twelve months. Most participants (72.6%) reported that their sexual partners were spouses or primary partners; 8.3% endorsed casual partners, and 5.8% said they visited sex workers. With casual partners and sex workers, 90.9% of respondents reported using condoms during the last sexual encounter, while only 32.1% of participants reported using condoms with their spouse or main partner. Average age at first sexual intercourse was 19.3 years (SD = 2.4), and it was slightly higher among participants from remote areas, 20 years (SD = 2.8), than those from urban areas, 19 years (SD = 2.2), *p* = 0.01. While overall 31.2% of participants reported using condoms during the last twelve months with spouse/main partner (SD = 44.1), this number was significantly lower for urban participants (24%) compared with rural participants (49.1%) (*p* < 0.01).

**Table 3:** Sexual behavior among respondents

Characteristics	Urban area		Remote area		Total		*p* value
	n	%	n	%	n	%	
Ever having sexual intercourse (n = 232)	156	96.3	69	98.6	225	97.0	0.35^a^
Number of sexual partners in the last 12 months (N = 223)							
• None	36	22.6	7	10.9	43	19.3	0.12^a^
• One partner	103	64.8	45	70.3	148	66.4	
• Two partners	15	9.4	7	10.9	22	9.9	
• > Two partners	5	3.1	5	7.8	10	4.5	
Type of partners in the last 12 months (N = 241)							
• Spouse/main partners	116	69.5	59	79.7	175	72.6	0.10^a^
• Casual partners	12	7.2	8	11.0	20	8.3	0.33^a^
• Sex workers	10	6.0	4	5.4	14	5.8	0.86 ^a^
Using condom in the last sexual encounter with							
• Spouse/main partners	29	25.9	25	44.6	54	32.1	<0.01^a^
• Casual partners	16	88.9	14	93.3	30	90.9	0.66^a^
• Sex workers	12	100.0	8	80.0	20	90.9	0.10^a^
	X	SD	X	SD	X	SD	*p*-value
Age at first sexual intercourse	19.0	2.2	20.0	2.8	19.3	2.4	0.01^b^
Percentage of using condom during the last 12 months with							
• Spouse/main partners	24.0	40.0	48.5	49.1	31.2	44.1	<0.01^b^
• Casual partners	88.9	33.3	92.0	17.9	90.0	28.0	0.74^b^
• Sex workers	80.0	42.2	86.7	23.1	81.5	37.8	0.82^b^

^a^ *Chi-squared test*

^b^ *Mann-whitney test*

Table 4 highlights factors associated with sexual behaviors among respondents. Current drug users were more likely (OR = 3.72; 95% CI = 1.16–11.92; *p* < 0.05) to report having > = 2 sex partners. People who were anxious or depressed were less likely (OR = 0.05; 95%CI = 0.00–0.70; *p* < 0.05) than patients who were not anxious or depressed to report having > = 2 sex partners. Participants living in remote areas were more likely (OR-3.82; 95%CI = 1.19–12.27; *p* < 0.05) than participants in urban areas to have > = 2 sex partners.

**Table 4:** Factors associated with sexual risk behaviors among respondents

Characteristics	Having ≥ 2 sex partners	
	OR	95% CI
Education (vs < High school)		
High school	2.36*	0.85; 6.52
Employment (vs Unemployment)		
Other	2.33	0.82; 6.58
Marital (vs Single)		
Live with spouse/partner	0.39	0.13; 1.22
Divorced/widow	0.08**	0.01; 0.93
Location (Remote vs Urban area)	3.82**	1.19; 12.27
Anxiety/Depression (Yes vs No)	0.05**	0.00; 0.70
EQ index	0.01***	0.00; 0.17
Current drug use (Yes vs No)	3.72**	1.16; 11.92
Current smoking (Yes vs No)	3.33	0.79; 14.05

***p < 0.01

**p < 0.05

*p < 0.1

## Discussion

This study sheds important light on the sexual behaviors of MMT patients residing in mountainous areas in Northern Vietnam. The results of our study highlight several potential areas of intervention to improve the sexual health and well-being of MMT patients and their sexual partners.

Overall, our study found a low proportion of having multiple sex partners among MMT patients (14.4%). This finding was comparable to the previous study that 12.3% of MMT patients had multiple sex partners [16]. Our rate was lower compared to that in other high-risk populations in Vietnam such as HTC clients (approximately 40%), PWID without MMT (45.0%) [8] and men who have sex with men (46.0%) [22]. Additionally, in line with previous literature [16], most of patients generally reported sexual engagement with their primary spouse/partner (72.6%), while few participants reported visiting sex workers (5.8%) or having casual sex (8.3%). Compared to other MMT populations in United States or Iran, the rate of having sex with nonprimary partners were lower among Vietnamese MMT patients [23, 24]. It should be noted that because this topic is sensitive in Vietnam, the patients might underreport their sexual behaviors, leading to the low rate of sexual risk behavior in this study. However, this result could be explained that after a long period of treatment, patients were more likely to engage in stable partnerships with their spouse/main partners.

Furthermore, the findings indicated that the frequency of condom usage among MMT patients with casual partners and sex workers were high, Previous literature on MMT patients has revealed conflicting evidence with regards to condom use. Some studies have found that MMT programs increase condom use among patients [7, 25], while others have reported no impact on condom use or a lower level of condom use among patients on MMT than their non-MMT counterparts [26]. The result implies the positive effect of MMT on sexual behaviors in drug users [16, 20].

One important finding was that current drug use was shown a strongly positive association with multiple sexual partners. This finding is in line with previous studies that suggested illicit drug use as leading to risky sexual behaviors [27, 28, 29]. Using illicit opioid drug might decrease the perception of risky behaviors and the capacity to control these behaviors among MMT drug users; and thus, could facilitate patients to engage in the sexual risk behaviors [30]. It underlines the importance of medication adherence among MMT patients in the study settings. Being on MMT, adhering treatment and ceasing opiate drug use could lead to more stable sexual practices, fewer casual partners or visits to sex workers, and decreased risk of HIV/AIDS transmission [1]. In this study, we found that 13.4% patients were concurrent drug users, suggesting high needs of specific attention and counseling to diminish the likelihood of engaging sexual risk behaviors among these patients.

In our study, patients who reported having anxiety/ depression had significantly lower odds of having two or more sexual partners. Several previous studies have shown that anxiety, depression, and other psychiatric disorders cause sexual dysfunction in patients on methadone therapy [31, 32]. Sexual dysfunction might reduce the sexual desire and the intimate relations between MMT patients and their spouse/partners, and consequently, diminish the patients’ quality of life [33]. Nonetheless, our study shows that increased HRQoL was associated with lower odds of multiple sexual partners. It might be because improving physical and psychological functions could contribute to better family life among MMT patients, resulting in the decreased likelihood of engaging in risky sexual behavior [33].

Of note, we found that rural remote patients were more likely to have multiple sexual partners than their peers in the urban setting. Previously, males living in rural parts of Vietnam have been found to have higher likelihood of visiting sex workers because many sex workers in Vietnam come from rural parts of the country [34, 35]. Thus, they might have more exposure to multiple sexual partners. We ignored the practice of polygamy in our study because it is illegal in Vietnam.

There are several implications that arise from our study. In Vietnam, there is overall increased need for education about the importance of sexual health even if an individual, who had history of unsafe drug injection, is having sex with a primary spouse/partner. Educational efforts would focus on influencing health behaviors at the individual, interpersonal, and group or community levels. Because rural patients were more likely to report having multiple sex partners compared to urban patients, this suggests that there may be need for additional behavioral health education about sexual health and behaviors in rural parts of mountainous regions in northern Vietnam. Health education that considers the patient’s attitudes (behavioral beliefs), subjective norms (normative beliefs) would likely influence sexual risk behaviors. Previous work in other countries has highlighted the impact that behavioral health education can have in combination with MMT to reduce HIV risk taking behaviors [35]. In addition, there must be increased efforts to reduce drug use and continue MMT adherence among the patients. Furthermore, providing comprehensive care to ensure the good HRQOL of MMT patients plays an important role in decreasing one’s sexual risk-taking behaviors.

Our study has several limitations. First, participants provided self-reported data, which introduces the possibility of recall bias. In addition, we did not perform urine drug test to confirm the relapse of opioid drug use among patients. We also did not collect data about comorbidities and dose of methadone. Therefore, further studies should be elucidated to include these variables as predictors of sexual risk behaviors among MMT patients. Second, we used a small and convenience sample of patients in Tuyen Quang province in northern Vietnam. The results of our study may not be generalizable to other parts of Vietnam or to MMT patients in other countries. Because this is a cross-sectional study, we may not be able to make causal inferences. Finally, because of the sensitive nature of the questions we asked participants, they may not have felt comfortable answering honestly about their sexual behaviors.

## Conclusion

This study highlights a low proportion of having sexual risk behaviors among MMT patients in Vietnamese mountainous settings. Integrating education about safe sexual practices into MMT services, along with providing medical care and ensuring methadone treatment adherence, is an important component in HIV risk reduction for these patients who were at risk of unsafety sexual practices.

## Abbreviations

AIDS: Acquired Immune Deficiency Syndrome
EQ-5D-5 L: EuroQol – 5 dimensions – 5 levels
HIV: Human Immunodeficiency Virus
HRQOL: Health-related quality of life
HTC: HIV testing and counseling
IVDA: Intravenous drug abuse
MMT: Methadone maintenance treatment
PWID: People who inject drugs
